# Weather and Suicide: A Decade Analysis in the Five Largest Capital Cities of Colombia

**DOI:** 10.3390/ijerph15071313

**Published:** 2018-06-22

**Authors:** Julián Alfredo Fernández-Niño, Víctor Alfonso Flórez-García, Claudia Iveth Astudillo-García, Laura Andrea Rodríguez-Villamizar

**Affiliations:** 1 Departamento de Salud Pública, Universidad del Norte, Barranquilla (Atlántico) ZP 081007, Colombia; aninoj@uninorte.edu.co (J.A.F.-N.);vfloreza@uninorte.edu.co (V.A.F.-G.); 2 Servicios de Atención Psiquiátrica, Secretaría de Salud, Ciudad de México ZP 11410, Mexico; 3 Departamento de Salud Pública, Universidad Industrial de Santander, Bucaramanga (Santander) ZP 68001, Colombia; laurovi@uis.edu.co

**Keywords:** suicide, weather, seasons, mental health, Colombia

## Abstract

Historically, seasonal variations in suicide rates were thought to be associated with changes in weather. Most of this evidence however, is based on studies that were conducted in developed countries that are located outside the tropics. As such, it is necessary to examine this association in developing countries, such as Colombia, which do not experience marked seasons. In addition, it is important to adjust for the effect of holidays when analyzing this association as they have been reported to be a relevant confounding factor. Our objective was to estimate the association between daily suicide incidence among men and women in five major Colombian cities (Bogotá, Medellin, Cali, Barranquilla, and Bucaramanga) and daily temperature and rainfall. For this purpose, we conducted a multi-city, multi-temporal ecological study from 2005 to 2015, using data from the suicide mortality registries (provided by the National Administrative Department of Statistics). Daily measurements of the two weather variables were obtained from the official historical registry of the meteorological station at each city airport. We used these data to estimate conditional Poisson models for daily suicide counts, stratifying by sex and adjusting for holidays. Although we found that none of the weather variable estimators could reject the null hypothesis, we uncovered an association between suicide incidence and long weekends in the total suicide model (Incidence Rate Ratio (IRR): 1.19, 95% confidence interval (CI): 1.04–1.23). We found no evidence of association between weather variables and suicide in Colombia. Our study is based on daily observations and it provides evidence of absence of this association in a tropical country that does not experience marked seasons.

## 1. Introduction

Suicide is an important global public health problem that has been increasing in the last three decades [1]. Current estimates state that 75% of all suicides occur in low- and medium-income countries [2]. Suicide is a multifactorial event with diverse biological and sociocultural determinants, such as mental health, social support networks, physical health, and significant life events [3]. In addition, previous studies have suggested that seasonal changes in suicide rate could be associated with changes in weather, although, the majority of this data comes from developed countries outside of the tropics where changes in weather conditions are more pronounced within the span of a year [3,4].

In the last two decades, more advanced statistical analyses and improved information systems have helped to confirm peaks in suicide in countries that experience seasons, especially during the spring and summer periods [5,6]. In particular, it was suggested that these peaks are related to changes in temperature [7,8,9], which in turn, would subsequently cause an increase in the levels of serotonin (5-hydroxytryptamine or 5-HT) and lead to states of greater impulsiveness and aggressiveness during periods of hot weather [9]. However, these findings were based on divergent evidence in which peaks were also associated with low temperatures [10]. Moreover, some other studies found no association with any of the weather variables [11]. There are also contradictory studies regarding the relationship between suicide incidence and precipitation and relative humidity, albeit based on less evidence [8,12,13].

Changes in season and weather conditions usually correspond to periods of the year in which there are also changes in social activities, such as summer holidays and certain festivities; it is methodologically challenging to separate the potential effects of the two types of determinants [7]. For example, in countries that experience marked seasons, such as Italy, an increase in suicides has been reported during December and April, months that coincide with Christmas and Easter, respectively [13,14,15]. Similar results in which special dates are related with suicide have also been observed in developing countries, such as Mexico [16]. The changes in social dynamics that occur during such dates has been proposed as the underlying factor of this phenomenon [17]. In fact, psychopathological symptoms tend to increase during holidays as a result of the changes in social interactions and the disappointment of unmet psychological expectations [18,19,20]. For this reason, any study analyzing the potential effects of weather on suicide should consider holidays as a possible confounding factor in the analysis. 

Literature dealing with the link between weather and suicide is very limited for medium-income countries that do not experience seasons. This is true for Colombia, which apart from having a different sociocultural context, experiences less fluctuations in weather when compared to countries that are located outside the tropics. Accordingly, one hypothesis to be explored is that if there is a true association between weather variables and suicides, this association should be also present in cities with lower weather variance, and remain even after adjusting by festivities. Therefore, evaluating the association between weather and suicide in Colombia will deepen our understanding of this relationship and help to establish a more global hypothesis of the causal relationships behind this phenomenon.

Here, our objective was to estimate the association between weather variables (temperature and rainfall) and the daily incidence of suicide among men and women living in the five major cities of Colombia between 2005 and 2015. 

## 2. Materials and Methods 

### 2.1. Study Location

Colombia is located in the northwest region of South America, and is approximately equidistant from the two extremities of the American continent. It extends from 4°13′30′′ S to 12°27′46′′ N, and from 66°50′54′′ W to 79°0′23′′ W. With a total land area of 1,141,748 km^2^, Colombia is the fourth largest country of South America. As Colombia is located in the intertropical zone, it has a large variety of weathers and ecosystems. Having an equatorial location (the equator line crosses the south part of the country), Colombia does not have drastic changes of sunlight all year long, and seasonal variations are related only to rainy and dry periods. 

The temperature in Colombia remains stable all year long; therefore, differences across cities are principally attributed to differences in geographic location (altitude). These differences are associated with variations in vegetation across the entire country. As such, the country is divided into six geographical regions: the Andean, Caribbean, Pacific, Orinoco, Amazon, and Insular regions. Bogotá (D.C.), Medellin, and Bucaramanga are located in the Andean region, but due to their different altitudes, they experience fairly different weathers. Whereas, Bogotá has an annual average temperature of 14 °C, Medellin and Bucaramanga averages are higher, 22 °C and 23 °C, respectively. On the other hand, Barranquilla and Cali are located in the Caribbean and Pacific regions, respectively. While Barranquilla is located at an altitude of 18 m and has annual average temperature of 27 °C, Cali has values of 1018 m and 24 °C. Having these heterogeneous weather conditions between cities, there are no strong variations in temperature within cities over the year. 

### 2.2. Study Design

We conducted a multi-temporal, multi-city ecological study. The observational unit was the day by city from the 1st of January 2005 to the 31st of December 2015. We included the five capital cities of Colombia with the largest population (Bogotá, Medellin, Cali, Barranquilla, and Bucaramanga). In 2015, the population of these five cities accounted for approximately 30% of the total population of Colombia and 39.2% of the total urban population.

### 2.3. Variables and Data Sources

#### 2.3.1. Response Variables: Suicide Counts

For each city, we determined the number of suicides per day by counting the number of deaths whose cause had been classified as one of the International Classification of Diseases (ICD)-10 codes X60-X84 or Y87.0. This information was obtained from the mortality database of the National Department of Statistics (DANE) for the whole period of the study. The total population by city and year was obtained from the DANE population projections, and was used as exposure variable in the analyses. By assuming that the total population of a city does not change within the course of a year, we were able to compare the suicide counts between cities. 

#### 2.3.2. Independent Variables: Weather Variables

We obtained daily data for temperature in degrees Celsius (average) and rainfall in millimeters (total amount). For the whole period 2005–2015, these data were obtained from the official historical registry of the meteorological station at each city local airport; therefore, information was obtained from one meteorological station by city. The daily average was computed as the arithmetic mean of hourly data when 75% or more of hourly data were available. Overall, information was available for 92% and 91% of the days for temperature and rainfall, respectively. 

In addition, we considered holidays to be a principal confounding factor. As such, we generated an indicator variable for holidays that consisted of the following four categories: regular working day (reference category), weekday holiday, long weekend, and special day but without the day off. Given that these days change every year in Colombia, we used official historical calendars to manually recover this information.

### 2.4. Statistical Analyses

All of the weather variables and suicide counts were summarized using measures of central tendency (mean and median) and dispersion (standard deviation and interquartile range). The distribution of the daily suicide counts among men and women was assessed using the dispersion index test (VIT) [21] and the Bohning asymptotic test [22]. As this test rejected the null hypothesis of equidispersion, we reasonably supposed a Poisson distribution of suicides for our analyses. Additionally, the autocorrelation in suicides rates was verified for both sexes using partial autocorrelation graphs and the Wallis test. We did not reject the null hypothesis in all of the mentioned tests (*p* > 0.10), so we did not have evidence of a seasonal trend in suicide rates.

We use multi-city conditional Poisson models for the number of daily suicides using the yearly population (total population and population by sex) as the exposure variable, the weather parameters as the main independent variables, and holidays as the main confounding factor. We used Poisson models that were conditioned by time strata (grouping by day, month, and year) to control for the seasonality of suicide data. In these models, weather effects are estimated when considering the structure of the correlation that the observations would have on the same stratum of day of the week, month, and year [23], and considering the city as a fixed effect.

We did not find evidence of a non-linear relationship between any of the independent variables and the logarithm of the expected value of the suicides rates when these relationships were explored graphically or using quartiles of the weather variables. Therefore, we used the independent variables in a continuous scale. 

We used multiplicative terms between each independent variable and the city indicator to explore heterogeneity between cities. This enabled us to obtain adjusted estimates of the coefficients for each city, estimates that are equivalent to performing city-stratified analyses, but more efficient. 

Subsequently, as an additional analysis, by using the moving averages of temperature and rainfall over the last seven days in each city as the main independent variables, we examined potential lagged effects, explored from one up to seven days before each observation, for each independent variable. Finally, we created fixed Poisson models that explored the cumulative effect of each variable.

All of the adjusted models were stratified by sex, and all of the assumptions were verified. An association was considered to be statistically significant at an alpha of 0.05. All of the analyses were performed using STATA 12 (Stata Corporation, College Station, TX, USA). 

## 3. Results

**Figure 1:**
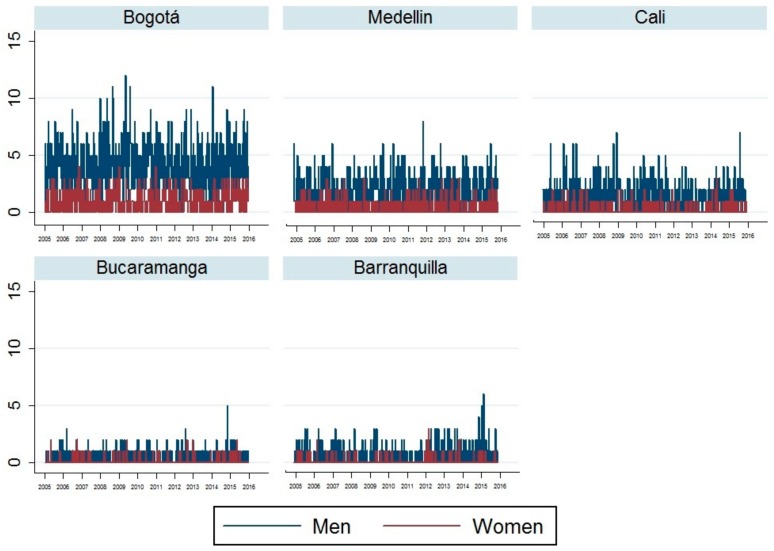
Weekly suicide count for the five main cities of Colombia, 2005–2015.

As a large quantity of the days in our study period were in fact, suicide-free days, we presented the suicide trend in men and women as a function of consecutive weeks (Figure 1). The average number of suicides per day for men and women, respectively, was 0.57 and 0.14 in Bogotá, 0.28 and 0.07 in Medellin, 0.21 and 0.04 in Cali, 0.06 and 0.02 in Bucaramanga, and finally, 0.09 and 0.01 in Barranquilla. 

**Figure 2:**
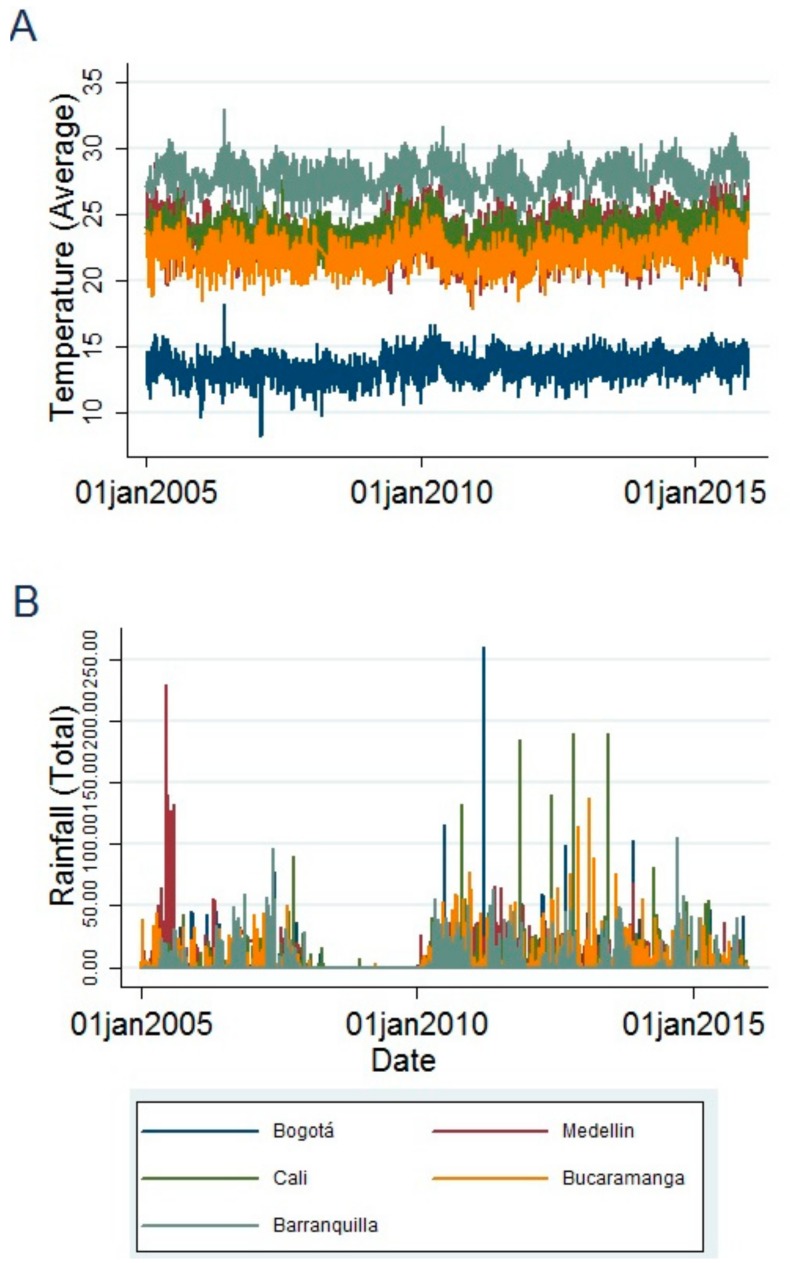
Weather variables of the five main cities of Colombia, 2005–2015. (**A**) Average daily temperature, (**B**) Daily rainfall.

Figure 2 displays the time series of the average daily temperature and rainfall for each of the five cities during the study period. This data, along with its central tendency and dispersion measures, is summarized in Table 1. Bogotá presents the lowest average temperature (13.67 °C), with a minimum value of 8.20 °C and a maximum of 18.20 °C. With respect to rainfall, Cali has the highest average value (3.98 mm), with a maximum of 185.66 mm.

The conditional Poisson models for suicide in men and women showed that temperature and rainfall do not present statistically significant associations with the number of suicides, neither in the general nor the sex-stratified models (Table 2). In contrast, with respect to the holiday indicator variable, we found that long weekends—in comparison to regular working days—not only have a statistically significant association with total suicides (Incidence Rate Ratio (IRR): 1.19, 95% confidence interval (CI): 1.04–1.23), but also with suicides in both sexes (IRR: 1.12, 95% CI: 0.97–1.31 for men and IRR: 1.51, 95% CI: 1.14–2.00 for women). However, this association is not observed for other categories of the same variable (i.e., weekday holiday or special day but without the day off). 

**Figure 3:**
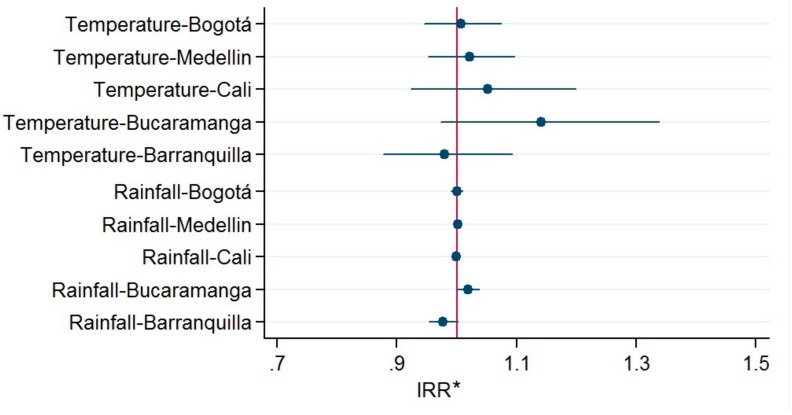
Estimators obtained for the weather variables in the five main cities of Colombia. Estimations were obtained using fixed-effect Poisson models and adjusted for the effect of holidays. *IRR can be interpreted as the average change in the suicide rate for each increment of one unit un each independent variable (a degree in the case if temperature, a cubic millimeter in the case of rainfall).

Figure 3 shows the graphical results of the Poisson models by city while considering the heterogeneity of each weather variable. For each of the five cities, we found that the null hypothesis could not be rejected for any of the analyzed weather variables. These results justify the use of pooled models (such as the ones that are proposed in this study) and they rule out the possibility that an association could have existed in only some of the cities. 

Finally, we did not find a statistically significant association between suicide counts and weather variables when considering their lagged (Table 3) and cumulative effects (Table 4) (i.e., the seven-day moving average).

## 4. Discussion

Here, we found no evidence supporting an association between weather (temperature and rainfall) and the daily suicide incidence among men and women of the five major cities of Colombia. However, we did find a statistically significant association between daily suicide counts and holidays, specifically long weekends. Furthermore, our results show that the association between suicide and holidays persists, even in the absence of marked variations in weather. This finding supports the fact that holidays are an important confounding factor to consider when studying the association between suicide and weather in any context. Controlling for holidays allows for us to focus on the potential explanatory mechanisms of the association between weather and suicide, such as geographic and cultural processes, more than just the associated physiological changes in the human body, as previous studies linking temperature effects on mental health have proposed [24,25]. The lack of evidence of a significant association between suicide and weather in this study might be explained by the fact that this relationship is modified in countries without drastic seasonal changes of weather, such as Colombia [26], or by potential confounding bias in previous studies. 

As there is currently a scarce amount of information available regarding this association in tropical or equatorial countries, our study brings to light important results. One study that analyzed this association in Sao Paulo—a city that is located in the southeast of Brazil close to the Tropic of Capricorn—found that variables, such as hours of sunlight and average temperature, had no effect on suicide during the 1996–2004 time period (based on official suicide registries) [27]. Another study conducted in Singapore [28]—an Asian country that is located one-degree north of the equator—neither found an association between maximum temperature and suicides when analyzing data from 1980 to 1989. The only exception to this was among Malaysian adolescents, for whom high temperatures appeared to account for about 58% of the variation in suicide incidence. It is also interesting to point out that upon stratifying the analyses, suicides committed by the Hindu population presented peaks in the months of April, September, and November, as well as during Deepavali, or the festival of the lights, which is the most important festival for the Hindu population, usually celebrated in the month of November. While the authors suggest that increased tensions due to extra expenses and forced family gatherings could be related to the rise in suicide during this festival, they also acknowledge that the existence of a true sociocultural association is not very clear [28]. 

The association between suicide and holidays has already been reported in studies that are conducted in Colombia. For example, while in the 2000–2010 time period there were a total of 24,882 suicides, the average daily number increased from an average of 6.2 to 8.0 when considering holidays. Furthermore, the largest number of suicides during this period was found to occur on the 1st of January and the 25th of December [29]. In fact, it is well known that psychopathological symptoms tend to increase around the holidays, including Christmas [18,19]. Such symptoms are enhanced during the holidays because during this time susceptible individuals experience an increase in failed social encounters, and consequently feel more frustrated, a feeling that is also consistent with an increase in dysphoric moods [20]. This susceptibility might be relevant, especially in people with a history of psychiatric disorders, as reported in a meta-analysis of 3275 suicides, where 87.3% of suicide cases had a history of psychiatric disorders [30].

An increase in the number of suicides during the holidays can also be the result of other causes, especially when considering that holidays are periods with a wide variety of stressors, including higher levels of alcohol consumption, changes in sleep rhythms, increased economic pressure, and more family conflicts [15]. Indeed, it has been reported that alcohol and drug consumption is related to 25–50% of all suicides [31], and that this risk is increased even further when the consumption is comorbid with other mental disorders [1].

These associations have also been widely explained by the broken promise effect of Gabennesh [32], in which spring, the weekends, and holidays are typically seen as positive events that can sometimes hold false promises (i.e., they are seen to offer more than what they can actually deliver). In such a way, these events can lead to a sense of hope and create expectations that are not met. This type of situation could be aggravated by a lack of social contacts [14] and/or by a more limited access to care and help [33], a situation that often occurs during holidays when services are closed or have reduced working hours [15]. Therefore, adding this negative emotional impact to the lack of social support could be a relevant risk factor, and have a greater impact in people with some affective disorder.

From the above discussion, it becomes evident that, when analyzing the relationship between suicide and weather variables, it is also important to take into account and isolate the effects of holidays and seasons. As holiday effect might interact with the weather variables, it becomes extremely difficult to distinguish between the two effects [12]. In our study, however, the geographical location of Colombia not only made it possible to isolate the seasonality effect, but also to explore the association in cities that are subjected to different weather [34]. 

We found no association between suicide and weather variables using data from the largest Colombian cities that helps to clarify this complex, heterogenic association in a country that does not experience marked seasonal variations. Regarding the quality of the suicide data, it is important to note that the data was obtained from the National Mortality Registry from the DANE. A quality assessment of the mortality registry was conducted by Cendales y Pardo [35] for the death certificates between 2002 and 2006 in Colombia and their results showed that 92.8% of the deaths were certified and coded correctly; the authors concluded with evidence that National Mortality Registry has a good quality in Colombia. Moreover, the underregistration of suicide seems to not be a problem in Colombia as previous studies have confirmed a steadily increase in the suicide registries, especially since 1998 [36]. In terms of weather data, the meteorological variables data was obtained from official reports for meteorological stations at local airports in all cities. Meteorological stations and registries are regulated by the Aeronáutica Civil de Colombia and follow quality system guideless, which support the quality of the registries. 

Therefore, our results provide important evidence that can be generalizable to other countries that are located close to the equator; however, more research in this geographical context is necessary to confirm the consistency of findings. Different from the countries where this association has been studied extensively, countries in the tropics only have dry and rainfall seasons. In the particular case of Colombia, the subdivision of the Andes mountain range in the national territory causes the formation of local and regional climates of high complexity and difficulty of prediction at different moments of time.

The main limitation of our study is its ecological design. As this type of study does not include individual variables, conclusions were interpreted for the population as a whole and cannot be extrapolated to the individual level. Another important limitation is that suicide records could be prone to errors at some stage of production, including collection, coding, data processing, or diagnosis [37]. This, in turn, would lead to the possibility of misclassified individuals. This type of scenario can lead to differential measurement errors. In addition, the small number of suicides is often a problem in these studies. Although we used data from the five largest Colombian cites, there is still a probability that type 2 error could explain the absence of any significant association. In this way, the low power of this study could be explained by the fact that Colombia, comparatively with other countries of the region, has a low suicide incidence [38], which could make difficult the finding of potential associations, especially those of low magnitude.

Finally, we use a very limited number of weather variables. In addition to the two weather variables that are evaluated here, the absolute humidity, the amount of sunlight radiation (Mj/m^2^) [39], and barometric pressure [24], were not available from the source data and should also be considered as potential variables that influence suicide. However, previous studies assessing weather and suicide have identified temperature and rainfall as the main weather factors that are associated with suicides [27,28], and our study included cities with different mean temperatures in the analysis. On the other hand, data concerning the exact location of a suicide (e.g., inside or outside of the home) should also be taken into account as this type of information clarifies whether weather conditions, such as high or low temperatures and rainfall, could have been a direct influencing factor [40]. 

In the future, more studies should be conducted to examine this association in countries that are located close to the equator or the tropics. By analyzing other counties that have different cultures and holidays, it would be possible to isolate the effect of holidays in the analysis. In addition, by considering risky behaviors that occur on holidays, whether a suicide takes place inside or outside of home, relevant social determinants, history, or psychiatric disorders, and the association with other elements of suicidal behavior, such as ideation, planning, or attempt, it would be possible to clarify further if the climatic variables are truly associated with suicide independently.

Finally, it would be also important to replicate studies conducted in different countries using similar methodologies, as the heterogeneity of the associations reported in other latitudes could be related to the use of different time frames, units of time (e.g., day or month), statistical methods, geographic locations, and socioeconomic conditions [24,26,37]. 

## 5. Conclusions

Overall, our results suggest that there is no association between weather variables and the incidence of suicide, at least in the five largest Colombian cities. However, we did find an association between suicide and holidays, specifically long weekends. As this association could be related to behavioral patterns and opportunities, it is crucial that prevention programs start to consider these elements, especially in the identification of high-risk groups (such as people with history of psychiatric disorders, mental health comorbidities, and risk behaviors, such as alcohol consumption) and promoting a monitoring of these factors, especially during holidays.

## Figures and Tables

**Table 1:** Distribution of the daily weather variables for the five main cities of Colombia.

	Mean	sd	p25	p50	p75	min	max
Bogotá							
Temperature	13.67	0.94	13.10	13.70	14.30	8.20	18.20
Rainfall	2.19	5.17	0.00	0.06	2.03	0.00	115.06
Medellin							
Temperature	23.49	1.41	22.60	23.44	24.30	18.10	31.60
Rainfall	3.30	11.19	0.00	0.00	2.03	0.00	229.62
Cali							
Temperature	24.07	1.38	23.10	24.07	25.10	19.90	29.00
Rainfall	3.98	13.01	0.00	0.00	0.89	0.00	185.66
Bucaramanga							
Temperature	22.71	1.33	21.80	22.70	23.57	17.80	31.60
Rainfall	2.17	6.82	0.00	0.00	0.93	0.00	95.50
Barranquilla							
Temperature	27.91	1.06	27.20	27.90	28.70	24.30	32.90
Rainfall	1.56	6.62	0.00	0.00	0.00	0.00	105.92

sd: standar deviation, p: percentile

**Table 2:** Conditional Poisson models for daily suicides among men and women in the five main Colombian cities, 2005–2015.

Variables	Men			Women			All		
	IRR	95% CI	*p*	IRR	95% CI	*p*	IRR	CI 95%	*p*
Temperature	1.01	0.97–1.05	0.51	1.06	0.98–1.16	0.15	1.02	0.99–1.06	0.21
Rainfall	1.00	1.00–1.00	0.61	1.00	0.99–1.01	0.73	1.00	1.00–1.00	0.74
Special dates (Ref: Regular working day)									
Weekday holiday	1.04	0.88–1.24	0.62	1.12	0.79–1.58	0.52	1.05	0.91–1.23	0.49
Long weekend	1.12	0.97–1.31	0.13	1.51	1.14–2.00	<0.01	1.19	1.04–1.23	0.01
Special day without the day off	1.10	0.81–1.51	0.54	1.49	0.83–2.67	2.67	1.17	0.88–1.54	0.27
*Temperature (deviation from mean)	1.01	0.97–1.06	0.55	1.08	0.98–1.18	0.11	1.03	0.99–1.07	0.21

* Estimations obtained from independent models using deviation of historical average temperature by city. In these models, other coefficients did not change. IRR = Incidence Rate Ratio. CI: confidence interval.

**Table 3:** Fixed Poisson models for the lagged effects of weather variables on daily suicides among men and women in five Colombian cities, 2005–2015.

	Men					Women				All			
	Lag	IRR	95% CI		*p*	IRR	95% CI		*p*	IRR	95% CI		*p*
**Temperature**	**L0**	0.99	0.92	1.07	0.84	1.05	0.88	1.25	0.58	1.00	0.93	1.08	0.92
	**L1**	1.06	0.96	1.16	0.26	0.94	0.77	1.14	0.52	1.03	0.95	1.13	0.43
	**L2**	0.99	0.90	1.08	0.76	1.10	0.89	1.35	0.37	1.00	0.92	1.09	0.92
	**L3**	0.96	0.87	1.06	0.40	1.14	0.93	1.40	0.21	0.99	0.91	1.08	0.83
	**L4**	1.05	0.96	1.15	0.31	0.88	0.72	1.08	0.22	1.02	0.94	1.11	0.66
	**L5**	1.02	0.93	1.12	0.67	1.11	0.91	1.36	0.29	1.04	0.95	1.13	0.41
	**L6**	0.93	0.85	1.02	0.11	0.99	0.81	1.20	0.88	0.94	0.86	1.02	0.13
	**L7**	1.03	0.95	1.12	0.45	0.84	0.71	1.00	0.05	1.00	0.93	1.07	0.90
**Rainfall**	**L0**	1.00	0.98	1.01	0.56	1.00	0.98	1.03	0.85	1.00	0.99	1.01	0.71
	**L1**	1.01	0.99	1.02	0.47	1.00	0.97	1.04	0.87	1.01	0.99	1.02	0.44
	**L2**	1.00	0.99	1.02	0.63	1.01	0.97	1.04	0.69	1.00	0.99	1.02	0.53
	**L3**	0.99	0.98	1.01	0.36	1.03	1.00	1.07	0.05	1.00	0.99	1.01	0.95
	**L4**	1.01	0.99	1.02	0.32	0.98	0.95	1.01	0.20	1.00	0.99	1.02	0.70
	**L5**	0.99	0.98	1.01	0.51	1.00	0.97	1.04	0.88	1.00	0.98	1.01	0.60
	**L6**	1.00	0.98	1.01	0.89	1.00	0.97	1.03	0.89	1.00	0.99	1.01	0.89
	**L7**	1.00	0.99	1.02	0.77	0.97	0.95	1.00	0.06	1.00	0.99	1.01	0.66

**Table 4:** Conditional Poisson models for the cumulative effects of weather variables on daily suicides among men and women in five Colombian cities, 2005–2015

	Men			Women			All		
	IRR	95% CI	*p*	IRR	95% CI	*p*	IRR	95% CI	*p*
**Temperature**	1.02	0.98–1.07	0.25	1.04	0.95–1.14	0.38	1.03	0.93–1.07	0.16
**Rainfall**	1.00	0.99–1.00	0.13	1.00	0.99–1.01	0.21	0.99	0.99–1.00	0.12

Estimations have been adjusted for the effect of holidays. The estimators represent changes with respect to the moving average of each weather variable in the last 7 days.
